# Chromatin regulated interchange between polycomb repressive complex 2 (PRC2)-Ezh2 and PRC2-Ezh1 complexes controls myogenin activation in skeletal muscle cells

**DOI:** 10.1186/1756-8935-4-16

**Published:** 2011-09-05

**Authors:** Lovorka Stojic, Zuzana Jasencakova, Carolina Prezioso, Alexandra Stützer, Beatrice Bodega, Diego Pasini, Rebecca Klingberg, Chiara Mozzetta, Raphael Margueron, Pier Lorenzo Puri, Dirk Schwarzer, Kristian Helin, Wolfgang Fischle, Valerio Orlando

**Affiliations:** 1 Dulbecco Telethon Institute, IRCCS Fondazione Santa Lucia, Rome, Italy; 2 Department of Oncology, University of Cambridge, Cancer Research UK Cambridge Research Institute, Cambridge, UK; 3 Biotech Research and Innovation Centre (BRIC), University of Copenhagen, Copenhagen, Denmark; 4 Laboratory of Chromatin Biochemistry, Max Planck Institute for Biophysical Chemistry, Göttingen, Germany; 5 Department of Biology and Genetics for Medical Sciences, University of Milan, Milan, Italy; 6 Department of Experimental Oncology, European Institute of Oncology, c/o IFOM-IEO Campus, Milan, Italy; 7 Leibniz-Institut fuer Molekulare Pharmakologie (FMP), Department of Chemical Biology/Protein Chemistry, Berlin, Germany; 8 Universitè Paris Diderot Paris 7, CNRS, Paris, France; 9 Curie Institute, Unité de Génétique et Biologie du Développement, Paris, France; 10 The Sanford Burnham Institute, La Jolla, CA, USA

## Abstract

**Background:**

Polycomb group (PcG) genes code for chromatin multiprotein complexes that are responsible for maintaining gene silencing of transcriptional programs during differentiation and in adult tissues. Despite the large amount of information on PcG function during development and cell identity homeostasis, little is known regarding the dynamics of PcG complexes and their role during terminal differentiation.

**Results:**

We show that two distinct polycomb repressive complex (PRC)2 complexes contribute to skeletal muscle cell differentiation: the PRC2-Ezh2 complex, which is bound to the *myogenin *(*MyoG*) promoter and *muscle creatine kinase *(*mCK*) enhancer in proliferating myoblasts, and the PRC2-Ezh1 complex, which replaces PRC2-Ezh2 on *MyoG *promoter in post-mitotic myotubes. Interestingly, the opposing dynamics of PRC2-Ezh2 and PRC2-Ezh1 at these muscle regulatory regions is differentially regulated at the chromatin level by Msk1 dependent methyl/phospho switch mechanism involving phosphorylation of serine 28 of the H3 histone (H3S28ph). While Msk1/H3S28ph is critical for the displacement of the PRC2-Ezh2 complex, this pathway does not influence the binding of PRC2-Ezh1 on the chromatin. Importantly, depletion of Ezh1 impairs muscle differentiation and the chromatin recruitment of MyoD to the *MyoG *promoter in differentiating myotubes. We propose that PRC2-Ezh1 is necessary for controlling the proper timing of *MyoG *transcriptional activation and thus, in contrast to PRC2-Ezh2, is required for myogenic differentiation.

**Conclusions:**

Our data reveal another important layer of epigenetic control orchestrating skeletal muscle cell terminal differentiation, and introduce a novel function of the PRC2-Ezh1 complex in promoter setting.

## Background

During development, differentiation programs require global rearrangements in repression and activation of lineage-specific genes. Chromatin-based epigenetic mechanisms ensure correct integration of developmental signals at gene regulatory regions, allowing the action of transcription factors and maintaining novel expression states in derived cell populations. Polycomb group (PcG) proteins are transcriptional repressors that remodel chromatin through epigenetic modifications that prevent changes in cell identity by maintaining transcription patterns, throughout development and in adulthood [1,2]. They comprise two major multiprotein complexes, polycomb repressive complex (PRC)-1 and PRC-2. PRC1 is the larger-sized complex that contains several polypeptides whose functions include ubiquitination of histone H2A at lysine 119 (H2AK119), chromatin compaction and regulation of the basal transcription machinery [3]. The core of the PRC2 complex is made up of three proteins, Suz12, Eed and Ezh2, the latter being the catalytic subunit that modifies histone H3 by trimethylation of lysine 27 (H3K27me3). Once H3K27me3 has been established, PRC2 is able to bind to this mark via the Eed subunit, which in turn activates the histone methyltransferase activity (HMT) of the complex [4,5]. This process allows maintenance of the repressive mark and its transmission to daughter cells [6]. Recently, it has been reported that in mammals HMTase Ezh2 can be replaced by another highly homologous polypeptide called Ezh1. However, whereas PRC2-Ezh2 catalyses H3K27me2/me3 and its knockdown affects global H3K27me2/me3 levels, PRC2-Ezh1 performs this function weakly [7,8]. Although Ezh1 depletion does not impact global H3K27me2/me3 levels, the PRC2-Ezh1 complex robustly represses transcription from chromatinised templates and compact chromatin [7]. Interestingly, while Ezh2 expression is closely associated with proliferation, Ezh1 is more abundant in non-proliferative adult organs, suggesting that these two PRC2 complexes may have different functions in dividing versus post-mitotic cells [9,10]. Thus, replacement of the Ezh2 subunit with Ezh1 appears to be developmentally regulated. To date, however, the function of Ezh1 in differentiating cells remains elusive.

Vertebrate skeletal muscle formation constitutes an interesting model system to study the epigenetic signals and molecular mechanisms that govern cellular differentiation [11,12]. Previous work revealed a crucial role of Ezh2 in skeletal muscle cell differentiation as its transcriptional and post-transcriptional downregulation is required to allow activation of muscle-specific genes [13,14]. During myogenic differentiation, extracellular signals are transduced into the nucleus by mitogen-activated protein kinases (MAPKs), p38 or extracellular signal-regulated kinase (ERK) [15,16]. The mitogen- and stress-activated protein kinases (Msk-1 and Msk-2), downstream targets of the p38 or ERK pathways [17], are responsible for the histone H3 phosphorylation at serine 28 (H3S28ph) and serine 10 (H3S10ph) [18,19]. Recent data show that an H3K27/H3S28 methyl/phospho switch mechanism regulates gene activation via PRC2 chromatin displacement during neuronal differentiation, stress response and mitogenic signalling [20,21]. If a similar mechanism is involved in muscle gene activation, allowing for PcG chromatin displacement, remains to be elucidated.

In the current work we report that two different PRC2 complexes contribute to skeletal muscle differentiation: PRC2-Ezh2, which is predominant in proliferating myoblasts, and PRC2-Ezh1, which contains Ezh1 but is devoid of Ezh2, and is specific for post-mitotic myotubes. Interestingly, these two PRC2 complexes are differentially associated with muscle regulatory regions. Indeed, while *muscle creatine kinase *(*mCK*) is a classic PRC2 target gene where its expression is associated with the displacement of PRC2-Ezh2 complex, *myogenin *(*MyoG*) shows a switch between PRC2-Ezh2/PRC2-Ezh1 complexes upon differentiation, suggesting a role of this dynamics in gene activation. In light of their different chromatin associations, we verified that a Msk1-dependent signalling that controls H3S28ph, is involved in the specific displacement of PRC2-Ezh2 from the *MyoG *and *mCK *regulatory regions, to result in muscle differentiation. This confirms the findings of previous reports that consider Ezh2 downregulation to be a necessary step in the myoblast-myotube transition [13,14]. Surprisingly, we found that the PRC2-Ezh1 complex is insensitive to the H3S28ph activation mark. Indeed, this complex regulates the proper timing of *MyoG *transcriptional activation via recruitment of MyoD transcriptional factor in post-mitotic myotubes.

Thus, our study reveals a novel important layer of PcG-mediated epigenetic regulation of skeletal muscle cell differentiation, in which the different dynamics and chromatin regulated switch between PRC2-Ezh2 and PRC2-Ezh1 complexes are coordinated to induce the transition from myoblast to myotube transcriptional programs.

## Results

### Two PRC2 complexes, PRC2-Ezh2 and PRC2-Ezh1, are present during myogenic differentiation

**Figure 1:**
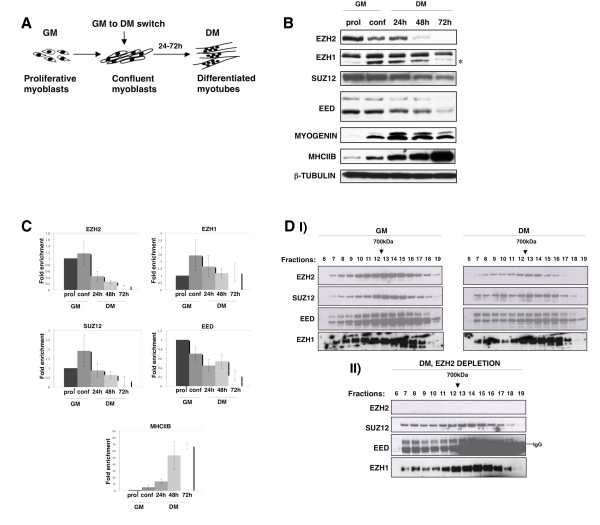
**Dynamics of PRC2-Ezh2 and PRC2-Ezh1 complexes during C2C12 skeletal muscle cell differentiation**. **(A) **Schematic representation of C2C12 skeletal muscle cell differentiation. Proliferative myoblasts at 80% confluency were induced to differentiate for 24-72 h by replacing growth medium (GM) with differentiating medium (DM). **(B) **Immunoblot of Ezh2, Ezh1, Suz12 and Eed from whole cell extracts of cells cultured as myoblasts in GM (prol = proliferative myoblasts at 50% confluence; conf = 80% confluent myoblasts) or as myotubes in DM (24 h, 48 h and 72 h after differentiation induction). Myogenin and myosin heavy chain IIB (MHCIIB) were used as muscle differentiation controls for the early and late stages of differentiation, respectively. β-Tubulin was used as a loading control. Asterisk indicates EZH1 unspecific band. Different bands of Eed represent the isoforms of this protein. **(C) **Expression levels of *Ezh2, Ezh1, Suz12 and Eed *were measured by real-time PCR in myoblasts grown in GM or DM, for 24 h, 48 h or 72 h after induction of differentiation. *MHCIIB *was used as a muscle differentiation control. The transcription levels were normalised to *Gapdh *expression and represent the mean of three independent experiments ± SD. Fold enrichment was calculated in comparison to myoblasts in GM. **(D) (I) **Size exclusion chromatography of nuclear extracts prepared from myoblasts in GM (left panel) or DM (72 h after differentiation induction) (right panel) showing coelution of Ezh2, Suz12, Eed and Ezh1 in high molecular weight fractions. The indicated fractions were analysed by immunoblot. **(II) **Ezh2 was immunodepleted from extracts prepared from cells cultured in DM (72 h after differentiation induction) and nuclear extract were analysed as described in (I).

It is known that decreasing levels of PcG Ezh2 protein activates the terminal myogenic program at the time of differentiation, thereby controlling skeletal muscle gene regulation [13,14]. However, the functions of other PRC2 components during the critical time of myoblast to myotube transition are not known. We sought to investigate the dynamics of PRC2 components during skeletal muscle differentiation of the C2C12 cell line. In this system, the replacement of growth medium (GM) by differentiation medium (DM) induces proliferative myoblasts to exit the cell cycle, to express muscle-specific genes, such as *MyoG*, *mCK *and *myosin heavy chain IIB *(*MHCIIB*), and subsequently to fuse into multinucleated terminally differentiated myotubes [22] (Figure 1A). Ezh2 protein was efficiently downregulated in differentiated cells (48 and 72 h after differentiation induction, Figure 1B), as previously reported [13,14], while Suz12 and Eed were still present in myotubes, although at lower levels. Curiously, Ezh1 levels remained constant throughout differentiation. A similar profile was obtained analysing the mRNA levels of these PRC2 components (Figure 1C). We performed the same analysis in human primary myoblasts triggered to differentiate into myotubes over the course of 8 days and in primary satellite cells isolated from adult mice. In these cells the pattern of PRC2 components was similar to that obtained in C2C12 cells, suggesting that the observed PRC2 dynamics is indeed a feature of skeletal muscle differentiation (Additional file 1). To investigate the composition of the PRC2 complex, we carried out size exclusion chromatography analyses of nuclear extracts from undifferentiated and differentiated C2C12 cells, followed by immunoblot of the eluted fractions for Ezh2, Suz12, Eed and Ezh1 components. The results showed that the majority of the four PRC2 proteins were eluted in a 700 kDa fraction (fraction 12-13) in both myoblasts and myotubes (Figure 1D, I), corresponding to the molecular weight of the PRC2 complex [23]. Since Ezh2 is degraded in myotubes (Figure 1B), we tested the possibility that an alternative PRC2 complex, formed by Suz12, Eed and Ezh1 and independent of Ezh2, could exist in post-mitotic cells. We therefore repeated the experiment using Ezh2 immunodepleted myotube extracts (Figure 1D, II). Interestingly, Suz12, Eed and Ezh1 subunits still coeluted at the same molecular weight. Taken together, these data suggest the existence of at least two PRC2 complexes in skeletal muscle cells, PRC2-Ezh2, predominant in proliferative myoblasts, and PRC2-Ezh1, more abundant in post-mitotic myotubes.

### PRC2-Ezh2 and PRC2-Ezh1 complexes are differentially associated with muscle gene regulatory regions

**Figure 2:**
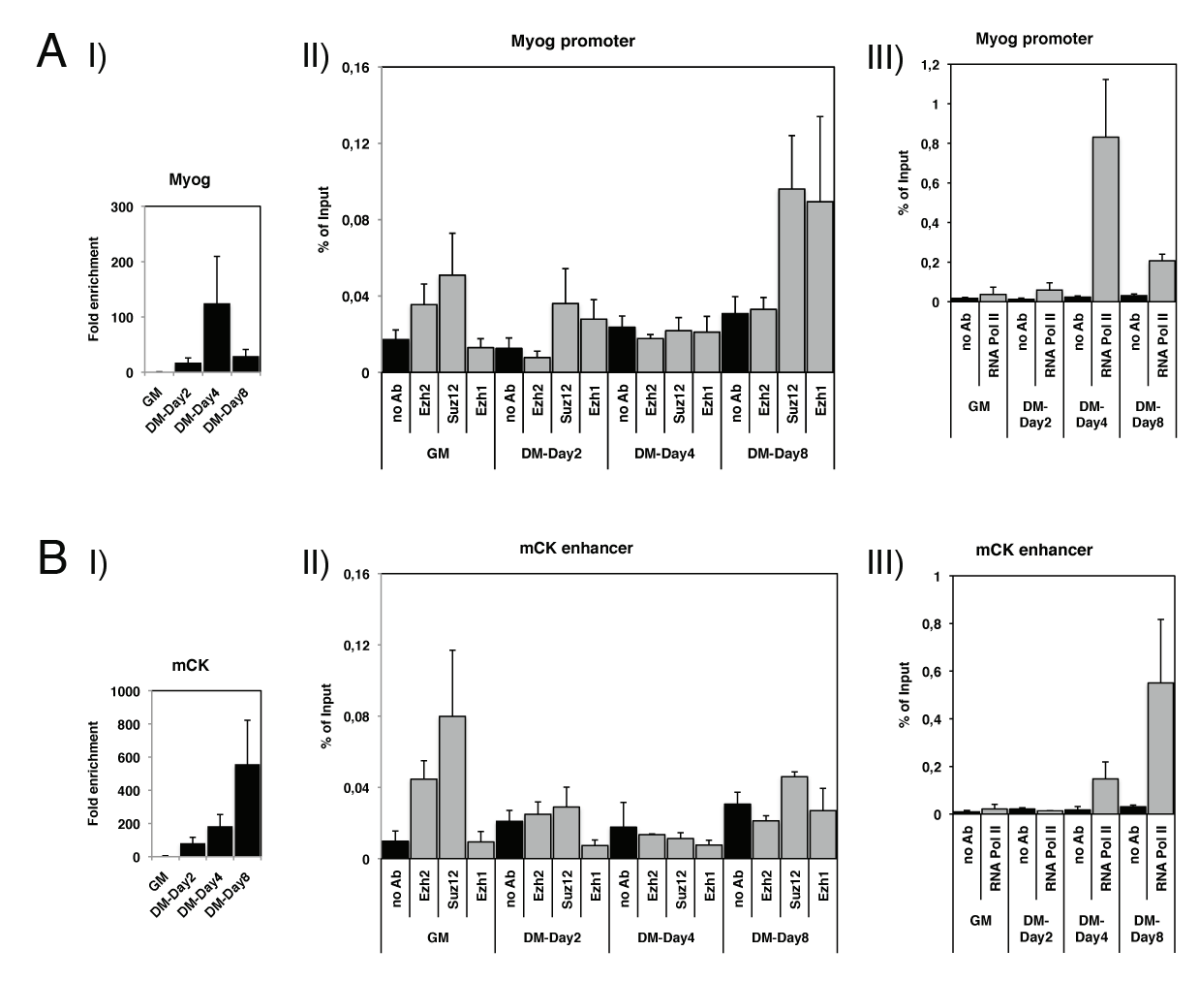
**Myogenin (MyoG) and muscle creatine kinase (mCK) muscle markers are differentially bound by PRC2-Ezh2 and PRC2-Ezh1 complexes in C2C12 cell lines**. **(A) (I) **Expression levels of *MyoG *were measured by real-time PCR in myoblasts grown in growth medium (GM) or differentiation medium (DM), 2, 4 or 8 days after induction of differentiation. The transcription levels were normalised to *Gapdh *expression and represent the mean of three independent experiments ± SD. Fold enrichment was calculated in comparison to myoblasts in GM. **(II, III) **Chromatin immunoprecipitation (ChIP) analysis of chromatin prepared from cells cultured in GM or in DM for 2, 4 and 8 days with Ezh2, Suz12, Ezh1 (II) and RNA polymerase II (RNA Pol II) (III) antibodies. The precipitated DNA fragments were subjected to real-time PCR analysis using primers designed within *MyoG *promoter. ChIP enrichments are presented as a percentage (%) of the input. Data are shown as an average of three independent experiments ± SD. **(B) (I) **Expression levels of *mCK *were measured by real-time PCR in myoblasts grown in GM or DM, 2, 4, or 8 days after induction of differentiation. The transcription levels were normalised to *Gapdh *expression and represent the mean of three independent experiments ± SD. Fold enrichment was calculated in comparison to myoblasts in GM. **(II, III) **ChIP analysis of chromatin prepared from cells cultured in GM or in DM for 2, 4 and 8 days with Ezh2, Suz12, Ezh1 (II) and RNA Pol II (III) antibodies. The precipitated DNA fragments were subjected to real-time PCR analysis using primers designed within *mCK *enhancer. ChIP enrichments are presented as percentage of the input. Data are shown as average of three independent experiments ± SD.

We then investigated the dynamics of the binding of PRC2-Ezh2 and PRC2-Ezh1 complexes to their targets, the *MyoG *promoter and *mCK *enhancer [13,14]. C2C12 cells were triggered to differentiate in low serum conditions over the course of 8 days, and chromatin immunoprecipitation (ChIP) experiments were performed before and after differentiation with antibodies against Ezh2, Suz12, Ezh1 and RNA polymerase II (RNA Pol II). This extended timecourse allowed us to observe the differences in the expression profiles of these two muscle-specific genes, *MyoG *and *mCK*. Indeed, *MyoG *was expressed in myocytes at day 2; levels peaked at day 4 and decreased at day 8, after fusion into polynucleated myotubes [24,25] (Figure 2A, I); in contrast, *mCK *levels increased throughout C2C12 differentiation [22] (Figure 2B, I). Ezh2 and Suz12 proteins (components of the PRC2-Ezh2 complex) were detected both on the *MyoG *promoter and *mCK *enhancer in undifferentiated myoblasts (Figure 2A, II and 2B, II). Although Suz12 remained bound to the *MyoG *promoter, Ezh1 replaced Ezh2 (PRC2-Ezh1) upon differentiation (DM day 2) (Figure 2A, II). These events correlated with RNA Pol II recruitment (Figure 2A, III). However, the levels of the binding of PRC2-Ezh1 and RNA Pol II at the *MyoG *promoter were inversely correlated during later stages of differentiation (DM days 4 and 8) (Figure 2A, II and 2III). Of note, we did not detect the PRC2-Ezh1 complex on the *mCK *enhancer in differentiating C2C12 cells (Figure 2B, II), whereas the recruitment of RNA Pol II progressively increased (Figure 2B, III).

Taken together, these results suggest that the binding of the PRC2-Ezh1 complex at the *MyoG *promoter in differentiating cells could play a role in the regulation of the proper transcriptional profile of this gene.

### A H3K27/H3S28 methyl/phospho switch regulates muscle gene activation via PRC2-Ezh2 chromatin displacement

**Figure 3:**
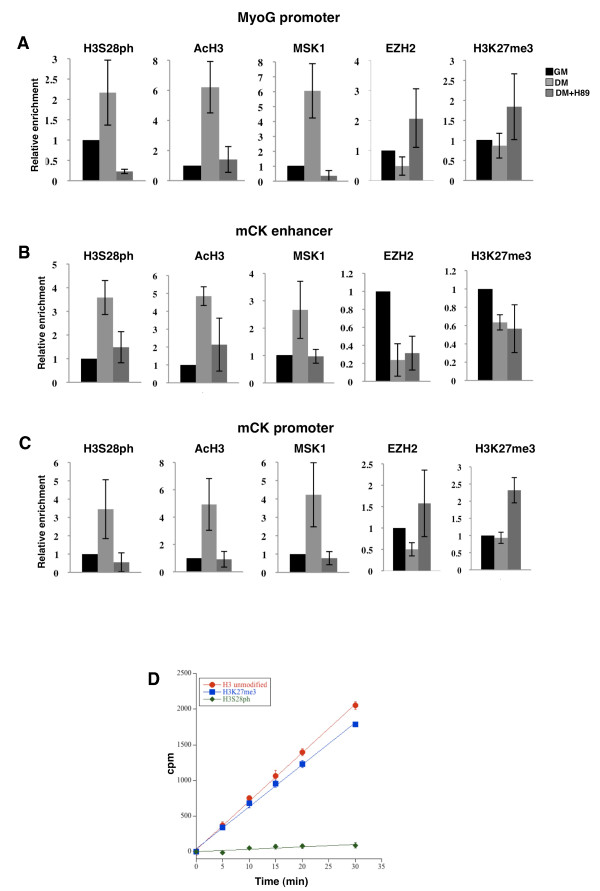
**Msk1-dependent H3S28 phosphorylation affects PRC2-Ezh2 chromatin displacement from muscle regulatory regions**. Chromatin immunoprecipitation (ChIP) analysis was performed on chromatin prepared from C2C12 cells cultured in growth medium (GM) or differentiation medium (DM) 48 h after induction of differentiation with or without Msk1 inhibitor H89 (5 μM) using histone H3 phosphorylation at serine 28 (H3S28ph), acetylated histone 3 (AcH3), Msk1, Ezh2 and H3K27me3 antibodies. The precipitated DNA fragments were subjected to real-time PCR analysis with primers amplifying the *myogenin *(*MyoG*) promoter **(A)**, *muscle creatine kinase *(*mCK*) enhancer **(B) **and *mCK *promoter **(C)**. ChIP values are presented as relative enrichments to myoblasts. Levels of H3S28ph, AcH3 and H3K27me3 were normalised to histone H3 density. Data represent the average of three independent experiments. Error bars represent SD. **(D) **Recombinant Msk1 was incubated with a histone H3 (residues 21-33) peptide either unmodified or modified with the K27me3 or S28ph, and a kinase assay was performed (cpm = counts per min).

Muscle gene activation requires the concerted recruitment of chromatin remodelling complexes, such as SWItch/Sucrose Non-Fermentable (SWI/SNF) [15] and the displacement of the PRC2-Ezh2 complex [13]. Our data, by showing that the PRC2-Ezh1 complex associates with the *MyoG *promoter, suggests evidence for an unexpected scenario in which signal-dependent changes in chromatin have to deal with two different PRC2 complexes. We decided to test the possibility that the previously reported H3K27/H3S28 methyl/phospho switch mechanism [20,21] could act at this level to regulate the PRC2-Ezh2 displacement during myogenic differentiation. We therefore analysed the binding of Msk1 and Ezh2 and their associated histone marks (H3S28ph and H3K27me3, respectively) at *MyoG *and *mCK *regulatory regions. Concomitant with the activation of these two genes, levels of H3S28ph and another active mark, acetylated histone 3 (AcH3), peaked at the *MyoG *promoter (Figure 3A) and *mCK *enhancer and promoter (Figure 3B, C) in myotubes. Enrichment of H3S28ph at these regions was associated with recruitment of Msk1 kinase (Figure 3A-C). Interestingly, in myotubes, an increase in H3S28ph correlated with the displacement of the PRC2-Ezh2 complex and the retention of H3K27me3 at *MyoG *(Figure 3A) and *mCK *promoter regions (Figure 3C). In contrast, at the *mCK *enhancer, loss of the PRC2-Ezh2 complex occurred simultaneously with H3S28ph enrichment and decrease in H3K27me3 during muscle differentiation (Figure 3B). Additionally, we analysed cells treated with H89, a compound known to inhibit Msk1 kinase activity [18,20,26]. Although H89 has been used at concentrations as high as 20 μM [18,27], lower doses (for example, 5 μM and 10 μM) were shown to inhibit Msk1 kinase more specifically [20,28]. Treatment with H89 impaired the establishment of the H3S28ph mark, the AcH3 mark and the recruitment of Msk1 kinase at *MyoG *promoter (Figure 3A), *mCK *enhancer (Figure 3B) and *mCK *promoter (Figure 3C) as well as activation of these genes (Additional file 2A-D). These events were accompanied by retention of PRC2-Ezh2 only at *MyoG *(Figure 3A) and *mCK *promoter regions (Figure 3C). In contrast, at *mCK *enhancer we did not detect PRC2-Ezh2 chromatin retention after H89 treatment (Figure 3B). The differences in Ezh2 binding between these two *mCK *regulatory regions and *MyoG *promoter could be explained by different degrees in H3K27me3 levels, in that this repressive mark increased upon H89 treatment at the *MyoG *(Figure 3A) and *mCK *promoters (Figure 3C) but not at the *mCK *enhancer (Figure 3B). Thus, the loss of the docking site H3K27me3 on the *mCK *enhancer could be sufficient to determine PRC2-Ezh2 chromatin displacement.

In light of the known role that Msk1 plays in the phosphorylation of H3S10 [18], we asked whether H3S10ph was also involved in muscle gene activation. However, because we did not observe any increase of this modification at the *MyoG *and *mCK *regulatory regions during muscle differentiation, we ruled out the possibility that H3S10ph functions in muscle gene activation (Additional file 2E). Furthermore, we examined whether Msk1 can phosphorylate H3S28 in an environment including pre-existing H3K27me3. Recombinant Msk1 kinase was incubated with a histone H3 (residues 21-33) peptide, which was either unmodified or modified with K27me3 or S28ph. Although the H3K27me3 substrate was phosphorylated under similar kinetic conditions as the unmodified peptide, no phosphorylation of the H3S28ph substrate was observed (Figure 3D), indicating that the serine 28 is the only residue phosphorylated by Msk1. Taken together, these data suggest that displacement of the PRC2-Ezh2 complex from *MyoG *and *mCK *promoters is regulated by a H3K27me3/H3S28ph switch via Msk1 recruitment onto chromatin.

### PRC2-Ezh2 and PRC2-Ezh1 chromatin dynamics are differentially regulated by a H3K27/H3S28 methyl/phospho switch

**Figure 4:**
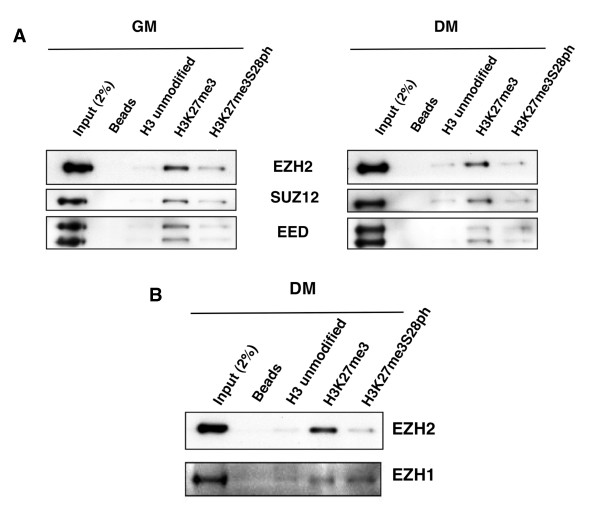
**H3K27me3/H3S28ph switch specifically influences the chromatin binding of the PRC2-Ezh2 complex but not PRC2-Ezh1**. **(A) **Nuclear extracts from C2C12 myoblasts (growth medium (GM)) and myotubes (differentiation medium (DM), 48 h after differentiation induction) were incubated with peptides representing unmodified H3, K27me3-H3 or the double modification K27me3S28ph-H3. Binding of Ezh2, Suz12 and Eed was tested by immunoblot. **(B) **Nuclear extracts from C2C12 cells cultured in DM, 48 h after induction of differentiation, were incubated with the same peptides as those listed in (A) and the binding of Ezh2 and Ezh1 was tested by immunoblot.

In order to provide direct mechanistic evidence for the involvement of the H3S28ph mark in the PRC2-Ezh2 chromatin displacement, we performed affinity-purification experiments using long histone H3 (residues 1-40) tail peptides, unmodified or modified with K27me3 or modified with the double mark K27me3S28ph, and we incubated them with nuclear extracts prepared from C2C12 myoblasts and myotubes. In agreement with earlier findings [4,6], Ezh2, Suz12 and Eed bound the H3K27me3 peptide (Figure 4A). Interestingly, interaction of all three PRC2 core components with the H3K27me3 docking site was significantly weakened in the presence of neighbouring H3S28ph (Figure 4A). The similar trend was observed when extracts prepared from undifferentiated myoblasts as well as from differentiated myotubes were used (Figure 4A). We therefore conclude that the ability of the PRC2-Ezh2 complex to bind H3K27me3 and to show sensitivity to H3S28ph is inherent to the complex, and is independent of differentiation. Since we observed that Ezh1 binding on the *MyoG *promoter upon differentiation (Figure 2A, II) occurs together with H3S28ph (Figure 3A), we next asked whether Ezh1 is retained on H3K27me3 even in the presence of the adjacent phosphorylated site. Comparable amounts of Ezh1 were bound to H3K27me3 and H3K27me3S28ph peptides from extracts of differentiated myotubes (Figure 4B). We conclude that Msk1-mediated phosphorylation of H3S28 impairs PRC2-Ezh2, but not PRC2-Ezh1 binding to its docking site, H3K27me3.

### Correct timing of *myogenin *transcriptional activation requires the PRC2-Ezh1 complex

**Figure 5:**
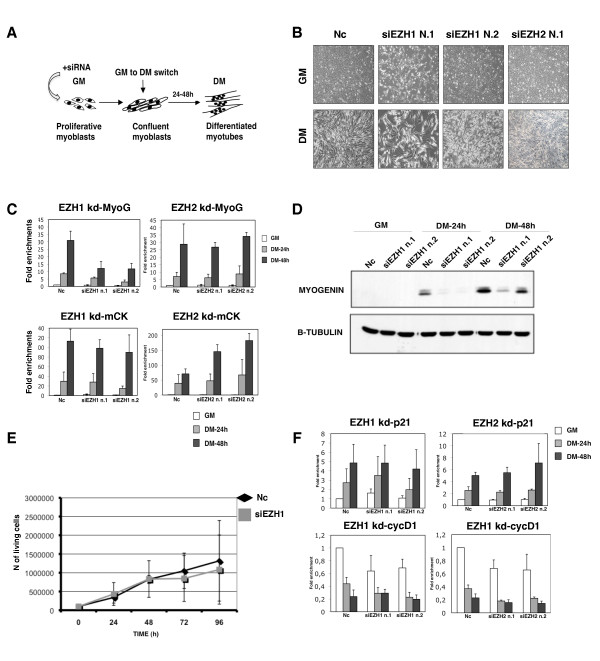
**Ezh1-depleted C2C12 cells show a delay of *myogenin *(*MyoG*) transcriptional activation**. **(A) **Schematic representation of the design of small interfering RNA (siRNA) knockdown experiments used in this study. **(B) **Myoblasts were transfected with non-targeting siRNA (Nc = negative control) or siRNA against Ezh1 and Ezh2. The effect of siRNA on cell morphology was analysed in growth medium (GM) (48 h after transfection) and in differentiation medium (DM) (48 h after differentiation induction) by phase-contrast microscopy. A single siRNA for Ezh2, representative of two different siRNAs, is shown. **(C) **Expression levels of *MyoG *and *muscle creatine kinase *(*mCK*) muscle markers were analysed by real-time PCR in GM and in DM. Transcription levels were normalised to *Gapdh *expression and represented as the average of three independent experiments ± SD. Fold enrichment was calculated in comparison to the negative control siRNA in GM. **(D) **Ezh1-depletion was performed as described in (A) and MyoG protein levels were analysed using whole cell extracts prepared from cells cultured in GM and DM, respectively. β-Tubulin served as a loading control. **(E) **Effect of Ezh1 depletion on cell proliferation. The cells were counted 24 h, 48 h, 72 h and 96 h after siRNA transfection. Ezh1 siRNA oligo no. 2 was used. Graph shows data from two independent experiments. Error bars represent the standard deviation (Nc = negative control). **(F) **Expression levels of *p21 *and *cyclin D1 *genes were analysed by real-time PCR in GM and in DM (24 h and 48 h after differentiation induction). The transcription levels were normalised to *Gapdh *expression and represented as the average of three independent experiments ± SD. Fold enrichment was calculated in comparison to the negative control siRNA in GM.

**Figure 6:**
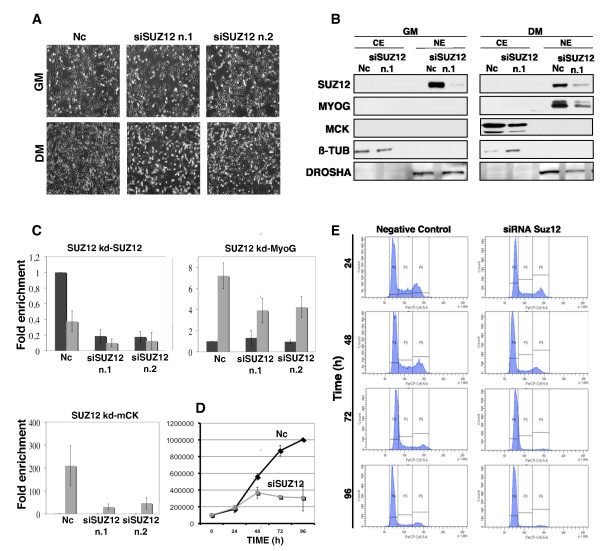
**Suz12 small interfering RNA (siRNA) impairs proliferation and differentiation in C2C12 cell lines**. **(A) **Myoblasts were transfected with non-targeting siRNA (Nc = negative control) or siRNA against Suz12. The effect of siRNA on cell morphology was analysed in growth medium (GM) (48 h after transfection) and in differentiation medium (DM) (48 h after differentiation induction) by phase-contrast microscopy. **(B) **Immunoblot of Suz12, myogenin (MyoG) and muscle creatine kinase (mCK) was performed after Suz12 depletion (oligo no. 1) using the experimental design described in Figure 5A and the protein levels were analysed in cells cultured in GM and in DM (48 h after differentiation induction), respectively. Nuclear (NE) and cytosolic (CE) extracts were used for the analysis, with β-tubulin serving as a cytosolic and Drosha as a nuclear extract control. **(C) **The efficiency of Suz12 siRNA and the expression levels of *MyoG *and *mCK *were tested by real-time PCR in GM and in DM (48 h after differentiation induction) in Suz12-depleted cells. The transcription levels were normalised to *Gapdh *expression and represented as the average of three independent experiments ± SD. Fold enrichment was calculated in comparison to the negative control siRNA in GM. **(D) **Effect of Suz12 depletion on cell proliferation. The cells were counted 24 h, 48 h, 72 h and 96 h after siRNA transfection. Suz12 siRNA oligo no. 1 was used. Graph shows data from three independent experiments. Error bars represent the standard deviation (Nc = negative control). **(E) **Cell cycle profiles were analysed by fluorescence-activated cell sorting (FACS) after Suz12 siRNA delivery at the same time timepoints as in (D). P4 gate represents G1 phase, P5 represents S phase and P6 represents G2 phase.

**Figure 7:**
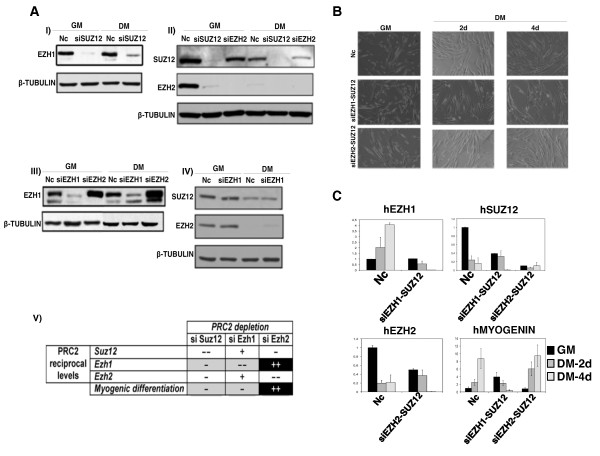
**Ezh1 is the unique PRC2 component required for skeletal muscle differentiation**. **(A) **Immunoblot analysis of Ezh1, Suz12 and Ezh2 levels in whole cell extracts prepared from C2C12 myoblasts cultured in growth medium (GM) and differentiation medium (DM), 48 h after differentiation induction, in Suz12-depleted **(I, II)**, Ezh2-depleted **(II**, **III) **and Ezh1-depleted **(III, IV) **cells. β-Tubulin served as a loading control. **(V) **Schematic representation of the muscle differentiation phenotypes detected after downregulation of single PRC2 components (Suz12, Ezh1 and Ezh2) and the impact on the protein levels of the remaining members of the complex. PRC2 reciprocal levels: - indicates that protein levels decline; -- indicates that protein levels strongly decline; + indicates that protein levels increase; ++ indicates that protein levels strongly increase. Myogenic differentiation: - indicates a delay in muscle differentiation; ++ indicates an acceleration of muscle differentiation. Phenotypes showing a delay in muscle differentiation are highlighted in grey, while phenotypes showing acceleration are in black. **(B) **Human myoblasts were transfected with non-targeting small interfering RNA (siRNA) (Nc = negative control) or siRNAs against *Ezh1-Suz12 *and *Ezh2-Suz12*. The effect of protein downregulation on cell morphology was analysed in GM (48 h after transfection) and in DM (2 and 4 days after differentiation induction) by phase-contrast microscopy. **(C) **The efficiency of each siRNA and the expression levels of *myogenin *(*MyoG*) were tested by real-time PCR in GM and in DM (2 days and 4 days after differentiation induction) in human myoblasts depleted for specific PRC2 components, as described in (B). The transcription levels were normalised to *Gapdh *expression and represented as the average of three independent experiments ± SD. Fold enrichment was calculated in comparison to the negative control siRNA in GM.

Our data show that the PRC2-Ezh1 complex is bound at the *MyoG *promoter upon gene activation (Figure 2A) and it is retained on H3K27me3 even in the presence of H3S28ph (Figure 4B). For these reasons, we explored the role of Ezh1 in *MyoG *regulation. We performed loss-of-function experiments in which C2C12 myoblasts were transiently transfected with two different small interfering RNAs (siRNAs) targeting Ezh1, and induced to differentiate for 48 h (Figure 5A), the temporal window in which *MyoG *is activated. As shown by phase contrast microscopy, Ezh1-depleted cells were not able to correctly differentiate (Figure 5B, central panels), while Ezh2-depleted cells differentiated normally in agreement with previously published data (Figure 5B, right panel) [13]. The efficiency of knockdown experiments is shown in Additional file 3. Ezh1-depleted cells displayed a delay in transcriptional activation of *MyoG *but not *mCK *(Figure 5C, left panels), while Ezh2-depleted cells did not show any decrease in *MyoG *and *mCK *expression (Figure 5C, right panels). The impairment in *MyoG *expression in Ezh1-depleted C2C12 cells was also confirmed at protein level (Figure 5D). Notably, a delay of *MyoG *transcriptional activation was also found in Ezh1-depleted human myoblasts and satellite cells (Additional file 4). In order to rule out the possibility that the muscle differentiation delay was due to an inability to switch off proliferation programs, we analysed the proliferative capability of C2C12 cells after Ezh1 knockdown. Ezh1-depleted myoblasts exhibited the same growth curve as the negative control (Figure 5E). Furthermore, p21 and cyclin D1 mRNA levels were not significantly affected either in Ezh1-depleted or in Ezh2-depleted cells (Figure 5F). Since Ezh1 was found in a complex with Suz12 and Eed in myotubes (Figure 1D, II), we performed the same knockdown approach targeting Suz12 in C2C12 cells (Figure 6), human myoblasts and satellite cells (Additional file 5). As revealed by phase contrast microscopy (Figure 6A Additional file 5A, C), a delay of muscle differentiation was detected after Suz12 depletion in each system, a result which was confirmed by lower protein (Figure 6B) and mRNA levels of MyoG and mCK muscle markers (Figure 6C Additional file 5B). In contrast to Ezh1 knockdown cells, the proliferation capability of Suz12-depleted C2C12 cells was impaired (Figure 6D). Indeed, flow cytometric analysis of the cell cycle revealed an accumulation of the cells in G1/S phase after only 48 h of treatment with Suz12 siRNA (Figure 6E), whereas the amount of apoptotic cells was comparable to the control cells (data not shown). These results, consistent with previously reported studies [29], may be explained by an autonomous cell cycle defect induced by the specific derepression of PRC2 target genes such as cytokines [30,31]. To further support the putative role of Ezh1 in controlling muscle differentiation, we compared the protein levels of the three PRC2 components, Ezh1, Ezh2 and Suz12, in each C2C12 siRNA experiment (Figure 7A). Interestingly, depletion of Suz12 resulted in the loss of both Ezh1 and Ezh2 proteins in myoblasts and myotubes (Figure 7A, I and 7II). Conversely, in Ezh2-depleted cells, we observed lower Suz12 and higher Ezh1 protein levels both in myoblasts and in myotubes (Figure 7A, II and 7III) while in Ezh1-depleted cells, we did not observe any change in Suz12 and Ezh2 protein levels (Figure 7A, IV). The results are summarised in Figure 7A, V: the loss of Ezh1 represents the only common factor in cells that were not able to differentiate (Suz12-depleted and Ezh1-depleted C2C12 cells). Indeed, cells that differentiate normally (Ezh2-depleted C2C12 cells) showed higher levels of Ezh1. In contrast, the levels of Suz12 and Ezh2 changed independently of the differentiation ability of the cells. The same results were obtained with a different experimental approach (Figure 7B, C). Human primary myoblasts were depleted for both Ezh1/Suz12 (PRC2-Ezh1 complex) or Ezh2/Suz12 (PRC2-Ezh2 complex) proteins. The depletion of both Ezh1 and Suz12 proteins impaired myogenic differentiation (Figure 7B). Indeed, lower MyoG mRNA levels were detected at 2 and 4 days after myogenic induction (Figure 7C). Conversely, cells depleted of both Suz12 and Ezh2 did not show any change in their differentiation ability (Figure 7B) and normal mRNA levels of MyoG were detected (Figure 7C). Thus, the process of myogenic differentiation is linked to the presence of Ezh1 or Ezh2 proteins, the components that distinguish PRC2-Ezh1 and PRC2-Ezh2 complexes.

### PRC2-Ezh1 is required for the recruitment of MyoD on *myogenin *promoter

**Figure 8:**
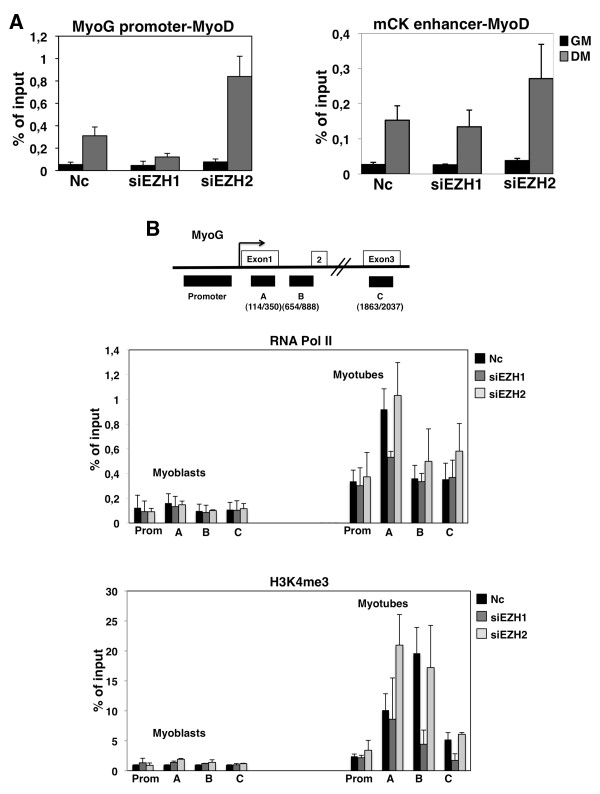
***Myogenin *(*MyoG*) transcriptional activation, via recruitment of MyoD, is regulated by the PRC2-Ezh1 complex**. Chromatin immunoprecipitation (ChIP) analysis was performed using chromatin prepared from C2C12 cultured in growth medium (GM) or differentiation medium (DM) (24 h from the induction of differentiation, the point at which *MyoG *is activated), after treatment with Ezh1 (oligo no. 2) and Ezh2 (oligo no. 2) small interfering RNA (siRNA), using MyoD antibody at the *MyoG *promoter and *muscle creatine kinase *(*mCK*) enhancer in (A) and RNA polymerase II (RNA Pol II) and H3K4me3 antibodies at different regulatory regions of *MyoG *gene in (B), as represented at the top of this panel. The precipitated DNA fragments were subjected to real-time PCR. Levels of H3K4me3 were normalised to histone H3 density. The values, shown as percentage of the input, represent the mean of three independent experiments. The mock was less than 0.01%. Error bars represent standard deviation.

Ezh1-depleted cells showed strong defects in the proper timing of *myogenin *transcriptional activation (Figure 5C), an event required for the initiation of myogenic differentiation. Since the MyoD transcription factor is considered one of the key elements involved in the *MyoG *activation process [32,33], we wondered if Ezh1 depletion could impair MyoD recruitment on the *MyoG *gene. Upon differentiation induction, lower levels of MyoD were detected in Ezh1-depleted myotubes (Figure 8A, left panel). The opposite effect was observed after Ezh2 knockdown (Figure 8A, left panel). Interestingly, we did not detect any decrease in MyoD binding at the *mCK *enhancer, for both the Ezh1-depleted and Ezh2-depleted cells (Figure 8A, right panel), suggesting that the delay in *MyoG *transcriptional activation could be a direct consequence of the impairment of MyoD recruitment at this particular gene. Moreover, after Ezh1 and Ezh2 depletion, we analysed the recruitment of RNA Pol II and the occurrence of active chromatin mark H3K4me3 on the *MyoG *promoter as well as on three different regions along the coding sequence of this gene (A, B and C). The binding of RNA Pol II was reduced at the coding region A, but not at the promoter, in the Ezh1 but not the Ezh2 knockdown (Figure 8B, upper panel), suggesting an impairment in the elongation step of the transcriptional process. Furthermore, only Ezh1-depleted cells displayed a reduction in the H3K4me3 active mark along the coding regions of *MyoG *(Figure 8B, lower panel). Taken together, these data demonstrate the importance of the role of the PRC2-Ezh1 complex in the correct timing of *MyoG *transcriptional activation through proper recruitment of MyoD on the *MyoG *promoter.

## Discussion

### Different dynamics of PRC2-Ezh2 and PRC2-Ezh1 complexes allow the correct timing of skeletal muscle gene transcriptional activation

PcG proteins contribute to differentiation through their ability to repress transcription of developmental regulators in committed cells, including skeletal muscle cell lines. Previous analysis of Ezh2 dynamics during myogenic differentiation has lead to a two-step activation model defining PcG-dependent muscle gene expression and cell differentiation [13]. However, a broad analysis of other PRC2 core components (Suz12 and Eed), including Ezh1, has not yet been attempted. Our data show that Ezh1 is the only PRC2 component that is maintained at constant levels during myogenic differentiation, while levels of Ezh2, Suz12 and Eed, to different extent, decrease from undifferentiated to differentiated states (Figure 1B, C and Additional file 1). We propose that skeletal muscle differentiation could be regulated by two distinct PRC2 complexes, PRC2-Ezh2 in myoblasts and PRC2-Ezh1 in myotubes. Existence of two partially redundant PRC2 complexes has been previously reported [7-10]. However, our data suggest that Ezh1 is more than just a substitute for Ezh2. Indeed, observations regarding the chromatin dynamics of the PRC2-Ezh1 complex on the *MyoG *promoter raise questions as to its functionality during skeletal muscle cell differentiation. Insight regarding the function of Ezh1 in skeletal muscle differentiation can be derived from the evidence that, unlike Ezh2 [13], Ezh1 is required for myogenic differentiation (Figure 5). In regard to this, we detected Ezh1 on the *MyoG *promoter when the gene is activated and RNA Pol II is recruited (Figure 2A). Indeed, Ezh1 depletion led to a delay of *MyoG *transcriptional activation due to the impairment of MyoD recruitment on the *MyoG *promoter (Figure 8). However, at the later stages of differentiation, the binding of Ezh1 and Suz12 (Figure 2A) could indicate that this complex has a role in the subsequent resilencing of *MyoG *in terminally differentiated myotubes. In agreement with this hypothesis, a recent report showed that *MyoG *upregulation during the initial stages of skeletal muscle differentiation is followed by subsequent repression [34]. Notably, *MyoG *is activated in the early stages of neurogenic muscle atrophy and failure in later downregulation is causally correlated with disease progression [35].

Surprisingly, our data showed that a PcG protein, such as Ezh1, is recruited on muscle specific gene when it is activated. Indeed, previous reports provided evidences that other PcG proteins bind actively transcribed genes [36,37]. The coexistence of active (AcH3, H3S28ph and H3K4me3) and repressive marks (H3K27me3) at the *MyoG *promoter could be similar to the bivalent domains of embryonic stem (ES) cells, as it has been shown that these domains are not limited to these cells [38]. Indeed, 10% to 20% of reported PcG target genes in ES cells are transcriptionally active [31,39]. The presence of PcG on active genes may be comparable to the presence of trithorax (trxG) proteins on repressed genes as this dual configuration of PcG and trxG proteins on active and repressed regions may provide a given gene with the flexibility to rapidly change its expression profile upon developmental or environmental stimuli.

As Ezh1 methyltransferase activity on histones is found to be modest [7], it will be interesting to investigate whether this PcG protein has targets in addition to histone H3, such as RNA Pol II enzyme. Indeed, a very recent report reveals that the C-terminal domain (CTD) of RNA Pol II is methylated by the coactivator-associated arginine methyltransferase 1 (CARM1) [40].

Future genome-wide analysis coupled to loss-of-function experiments will be required to address EZH1 function in myofibres.

### H3K27/H3S28 methyl/phospho switch mechanism is the basis of PRC2-Ezh2 target gene activation during myogenic differentiation

If PRC2-Ezh1 is required for the correct timing of *MyoG *transcriptional activation, removal of PRC2-Ezh2 from this gene would be necessary to guarantee its activation. One way of doing this would be to reduce intracellular PcG levels. In regard to this, Juan *et al*. [14] provided evidence that miR-214 regulates Ezh2 protein levels in skeletal muscle and ES cells. Recent studies raise interesting questions concerning the assumption that PcG derepression must be accompanied by the loss of the H3K27me3 repressive mark. Seenundun and coworkers [41] showed that the histone demethylase UTX is targeted to muscle-specific genes by the transcriptional activator Six4 to mediate removal of the repressive H3K27me3 mark during myogenesis. Recent reports suggest that demethylation of H3K27 may not be the only mechanism for derepression of PcG target genes [20,21]. A novel mechanism regulating PcG displacement from chromatin has been identified, in which phosphorylation of H3S28, via mitogen and stress-activated kinases Msk1 and 2, is able to neutralise the H3K27me3 repressive mark to result in PRC2 removal and gene activation [20,21]. Our data show that a similar mechanism appears to operate in differentiating myoblasts, in which Msk1 regulates a H3K27/H3S28 methyl/phospho switch to allow removal of the PRC2-Ezh2 complex and muscle gene activation (Figure 3). Notably, our *in vitro *experiments indicate that the Msk1-methyl/phospho switch pathway is specific to the PRC2-Ezh2 complex, while it appears that PRC2-Ezh1 is not regulated by this mechanism (Figure 4). Our ChIP analysis shows that the H3K27me3 mark is not alternative to H3S28ph and we can detect them independently. The *in vivo *presence of a phospho group at H3S28 may interfere with epitope recognition of H3K27me3 antibodies, raising potential concerns about the interpretation of the existing H3K27me3 ChIP genome-wide database [11]. In our ChIP experiments we did not encounter this problem as H3K27me3 was efficiently detected, even in the presence of adjacent H3S28ph mark. Previous studies suggest that PRC2 function is required during S-phase to guarantee maintenance of silenced state [6]. A recent genome-wide analysis of histone modifications performed in C2C12 myotubes revealed that the H3K27me3 mark on repressed non-muscle genes is not associated with PRC2, but with PRC1 complexes [42]. Thus, the function of the PRC2 complex in post-mitotic myotubes may not be linked to the maintenance of the H3K27me3 mark. Indeed, our data suggest that the PRC2-Ezh1 complex, and in particular the Ezh1 subunit, is required for proper *MyoG *activation when H3K27me3 mark is not removed, suggesting that Ezh1 function is linked to promoter setting of terminally differentiating cells. Future experiments will be required to test the hypothesis that while some genes are permanently inactive and do not require PRC2-Ezh2 activity once cells have stopped proliferating, other genes remain active and maintain their competence to resilence by using chromatin bound PRC2-Ezh1, as a security measure.

## Conclusions

Our work addresses the role of PRC2 complexes during skeletal muscle cell differentiation.

We report that two different PRC2 complexes, PRC2-Ezh2 and PRC2-Ezh1, are differentially associated with muscle gene regulatory regions and play distinct roles in the terminal differentiation process. We show that as Ezh2 is removed from *MyoG *and *mCK*, high levels of Ezh1 persist in differentiating muscle cells and PRC2-Ezh1 is recruited at *MyoG*, a step that is essential for activation of the early myogenic program. These events are required for regulation of the correct timing of *MyoG *transcriptional activation, and loss of Ezh1 affects recruitment of the MyoD transcription factor on its promoter in post-mitotic myotubes. Further, we report that Msk1-signalling controls H3S28ph and is involved in the specific displacement of PRC2-Ezh2 from muscle regulatory regions, triggering muscle gene activation and thereby muscle cell terminal differentiation. Consistent with its role involving *MyoG *transcriptional activation, we show that the PRC2-Ezh1 complex is insensitive to the H3S28ph activation mark. Thus, our study reveals a novel important layer of PcG-mediated epigenetic regulation of skeletal muscle cell differentiation, in which the coordinated different dynamics and chromatin-regulated switch between PRC2-Ezh2 and PRC2-Ezh1 complexes are required to initiate the transition from myoblast to myotube transcriptional programs. Notably, our data suggest a novel and unexpected role for PRC2-Ezh1 in promoter setting. Further, based on published data concerning MyoG regulation in muscle fibres, we speculate that PRC2-Ezh1 may be required for subsequent developmentally regulated resilencing of *MyoG *and perhaps other skeletal muscle genes. Our study provides new epigenetic insights into the process of terminal differentiation, in which the regulated and coordinated chromatin dynamics of two PRC2 complexes is required for the correct timing of muscle gene activation and thereby muscle differentiation.

## Methods

### Cell lines and reagents

C2C12 mouse myoblasts cells (ATCC, Manassas, VA, USA) were cultured in Dulbecco's modified Eagle medium (DMEM) supplemented with penicillin/streptomycin and 10% fetal bovine serum (FBS) (Euroclone, Devon, UK). Differentiation was induced when cells reached approximately 80% confluency using DMEM containing ITS media supplement (Sigma, St Louis, MO, USA) or 2% horse serum (HS) (Euroclone). Human primary myoblasts from healthy donors were obtained from the Telethon BioBank (Neuromuscular Diseases and Neuroimmunology Unit, Muscle Cell Biology Laboratory, C Besta Neurological Institute). The cell lines were cultured in DMEM supplemented with 20% FBS (Lonza, Basel, Switzerland), insulin 10 mg/ml, human fibroblast growth factor (hFGF) 25 ng/ml, human epidermal growth factor (hEGF) 10 ng/ml (proliferating medium), and then induced to differentiate by means of DMEM supplemented with 2% HS (differentiating medium). H89 (Alexis Corporation, Farmingdale, NY, USA) was replaced every 24 h.

### Satellite cell isolation and culture

Single muscle fibres were isolated by standard procedures. In brief, the hind limb muscles were digested with collagenase and single myofibres were cultured in GM1 (DMEM supplemented with 10% HS (GIBCO, Invitrogen, Carlsbad, CA, USA), 0.5% chick embryo extract (MP Biomedicals, Illkirch, France), and penicillin-streptomycin (GIBCO)) at 37°C in suspension for 72 h, and then plated on matrigel (Sigma, 1 mg/ml ECM gel)-coated dishes for satellite cell culture. Then, 3 days later, the fibres were removed and the medium replaced with proliferation medium (GM2: 20% FBS, 10% horse serum, 1% chick embryo extract in DMEM). After 4-5 days, the medium was replaced with differentiation medium (DM: 2% HS and 0.5% chick embryo extract in DMEM).

### RNA isolation and quantitative real-time PCR

RNA was extracted from cells using TriReagent (Sigma) according to the manufacturer's instructions. cDNA synthesis was performed using the QuantiTect reverse transcription kit (Qiagen, Hilden, Germany). Quantitative real-time PCR reactions were performed in triplicate using QuantiTect SYBR Green master mix (Qiagen) on a DNA Engine Opticon 2 machine (MJ Research) controlled by Opticon Monitor 2 software. C(T) values were calculated by Opticon Monitor 2 software. Gapdh, MHCIIB and mCK primers have been previously described [13]. The remaining primer sequences are available upon request.

### RNA interference

C2C12 cell line and satellite cells: siRNA EZH1 no. 1 (SI00997766), siRNA EZH1 no. 2 (SI00997773), siRNA SUZ12 no. 1 (SI01438416), siRNA SUZ12 no. 2 (SI01438402), as well as negative control siRNA (scrambled sequence not targeting mouse genome, 1027313) were purchased from Qiagen. The remaining siRNA sequences are as follows: siRNA Ezh2 no. 1: AAGGAAAGAACTGAAACTTA; siRNA Ezh2 no. 2: AAGCTGAAGCCTCCATGTTTA.

Cells were transfected with HiPerfect (Qiagen) following the manufacturer's instructions. At 48 h after transfection the cells were induced to differentiate and collected at the indicated timepoints. All siRNAs were used at a final concentration of 20 nM.

Human myoblasts: cells were transfected with DharmaFECT (Thermo-Scientific, Waltham, Massachusetts, USA) following the manufacturer's instructions. At 48 h after transfection the cells were induced to differentiate and collected at the indicated timepoints. All siRNAs were used at a final concentration of 6 nM (Ambion/Applied Biosystems, USA). The siRNA sequences are available upon request.

### Cell lysis and immunoblot

Cells were harvested and washed twice with PBS. Cell lysis of total cell extracts was performed on ice in 50 mM Tris-HCl pH 8, 125 mM NaCl, 1% NP-40, 2 mM ethylenediaminetetra-acetic acid (EDTA), 1 mM phenylmethylsulfonyl fluoride (PMSF) and protease inhibitory cocktail (Roche, Madison, WI, USA)) for 25 min. Insoluble material was pelleted by centrifugation at 16000 g for 3 min at 4°C. Protein concentration was determined using the Bradford assay (Bio-Rad, Hercules, CA, USA). The proteins were denatured, reduced, separated by SDS-PAGE and transferred to nitrocellulose transfer membrane. The membranes were blocked with 5% non-fat dry milk in Tris-buffered saline (TBS) supplemented with 0.1% Tween (Sigma) (TBST) and incubated with primary antibodies overnight at 4°C. Following three washes with TBST, membranes were incubated with the peroxidase-conjugated secondary antibody, in TBST with 2.5% non-fat dry milk, and immunoreactive proteins were detected using Supersignal West Dura HRP Detection Kit (Thermo-Scientific). For cytoplasmic and nuclear extracts preparation the cells were resuspended first in buffer A (10 mM 4-(2-hydroxyethyl)-1-piperazine-ethanesulfonic acid (Hepes), pH 7.9, 10 mM KCl, 0.1 mM EDTA and 0.1 mM ethylene glycol tetra-acetic acid (EGTA)) supplemented with protease inhibitory cocktail (Roche), 1 mM dithiothreitol (DTT) and 1 mM PMSF. After incubation on ice for 10 min, NP-40 was added to a final concentration of 0.5% and the samples were vortexed for 5 s. Nuclei were pelleted at 13,200 rpm for 10 s and the cytoplasmic proteins were collected. The pellet was then washed five times with buffer A and resuspended in buffer C (20 mM Hepes pH 7.9, 400 mM NaCl, 1 mM EDTA, 1 mM EGTA, 1 mM DTT, protease inhibitory cocktail (Roche) and 1 mM PMSF). After 10 min on ice, the samples were sonicated and centrifuged at 13,200 rpm for 10 min and nuclear proteins were collected.

### Chromatin immunoprecipitation

ChIP was performed as previously described (Breiling A and Orlando V, doi:10.1101/pdb.prot4560, with adaptations) using a crosslinking time of 10 min. Antibodies were coupled to Dynal magnetic beads (Invitrogen) by overnight incubation at 4°C. The following day, chromatin was added to antibody-bead complexes and incubated overnight at 4°C. The bound complexes were washed twice in Low Salt Solution, twice in High Salt Solution, once in LiCl and once in Tris/EDTA (TE) buffer. DNA was extracted from beads by standard phenol/chloroform extraction, precipitated and resuspended in 30 μl TE. To quantify the results, quantitative (q)PCR reactions were performed in triplicate (precipitated DNA samples as well as serially diluted input DNA) using QuantiTect SYBR Green master mix (Qiagen) on a DNA Engine Opticon 2 machine (MJ Research) controlled by Opticon Monitor 2 software. C(T) values were calculated by Opticon Monitor 2 software. To calculate relative enrichment the signal from the control immunoprecipitation experiment (Mock) was subtracted from that observed with the antibody of interest. Myoblasts values (GM) were set as 1 and values from differentiated cells in DM with or without inhibitor display relative enrichment or reduction to those observed in GM. ChIP primers are available upon request.

### Antibodies

For immunoblot: EZH2 (3147) was from Cell Signaling (Danvers, MA, USA). SUZ12 (46264), MyoG (12732), and MHCIIB (2064) were from Santa Cruz (Santa Cruz, CA, USA). β-Tubulin (T0198) was from Sigma. mCK antibody was kindly provided by Hidenori Ito (Aichi Human Service Center, Kasugai, Aichi, Japan). Ezh1 and EED antibodies were previously characterised ([7], [23]). For ChIP: H3K4me3 (8580), RNA polymerase II (5408) and SUZ12 (12073) were from Abcam (Cambridge, UK) while Ezh2 (07-400), H3K27me3 (07-449), H3S28ph (07-145), H3S10ph (05-817) and Acetyl H3 (06-599) were purchased from Millipore (Billerica, Massachusetts, USA). MSK1 (9392, 25417) and MyoD (760) were from Santa Cruz.

### Size exclusion chromatography

Size exclusion chromatography was performed using C2C12 cell nuclear extracts on a Superose 6 PC 3.2/30 gel filtration column (GE Healthcare, Little Chalfont, UK) using an AEKTA purifier system (GE Healthcare) in IP (300) buffer (50 mM Tris-HCl at pH 7.5, 300 mM NaCl, 5% glycerol, 0.2% Igepal (Sigma), Aprotinin, Leupeptin, 100 mM PMSF, 1 mM DTT). Immunodepletion was performed as described [43]. Briefly, protein extracts were subjected to five serial depletions within 24 h at 4°C using the AC22 EZH2 monoclonal antibody [44] precoupled to Protein-A beads.

### Histone tail peptides

Histone H3 peptides were synthesised in unmodified and modified form using Fmoc (*N*-(9-fluorenyl)methoxycarbonyl)-based solid-phase synthesis. Peptides used for kinase assays corresponded to amino acids 21-33 of H3 containing an artificial Y at the C-terminus: H3 unmodified, ATKAARKSAPATGY; H3K27me3, ATKAARK(me3)SAPATGY; H3S28ph, ATKAARKS(ph)APATGY. Peptides used for precipitation experiments corresponded to amino acids 1-40 of H3 and contained a C-terminal non-native YCK sequence with the lysine biotinylated at the e-amino group: H3 unmodified, ARTKQTARKSTGGKAPRKQLATKAARKSAPATGGVKKPHR-YCK (biotin); H3K27me3, ARTKQTARKSTGGKAPRKQLATKAARK(me3)SAPATGGVKKPHR-YCK (biotin); H3K27me3S28ph, ARTKQTARKSTGGKAPRKQLATKAARK(me3)S(ph)APATGGVKKPHR-YCK (biotin).

### *In vitro *peptide kinase assay

Recombinant MSK1 (Millipore) was used to phosphorylate H3 histone tail peptides (21-33). Kinase assays were performed according to the manufacturer's protocol by incubating 15 ng of MSK1 with 1 μg of peptide for 30 min at 30°C. The reaction was stopped by adding 0.5% phosphoric acid, spotted on P81 paper and washed three times with 0.5% phosphoric acid and once with acetone. Filter circles were air dried and counted in a scintillation counter.

### Peptide affinity purification

For preparation of nuclear extracts, cells were lysed in buffer A (10 mM Hepes-KOH pH 7.8, 60 mM KCl, 1 mM EDTA, 1 mM DTT, protease inhibitor cocktail (Roche), 0.075% NP-40). After incubation on ice for 15 min, nuclei were pelleted and washed once with buffer A without NP-40. The nuclear pellet was suspended in buffer B (20 mM Tris pH 8.0, 300 mM NaCl, 1.5 mM MgCl_2_, 0.2 mM EDTA, 25% glycerol, 1 mM DTT, protease inhibitor cocktail, PhosSTOP (Roche)) and sonicated on ice in a Branson Sonifier (duty cycle 20%, output 7.5). Extract was left on ice for 30 min before centrifugation for 15 min at 16,000 *g*. The supernatant was supplemented with 0.1% Triton X-100 and used for precipitation experiments.

For H3 peptide precipitation experiments, 10 μg of biotinylated histone peptides (1-40) were coupled to 50 μl streptavidin-coated paramagnetic beads in PBS/bovine serum albumin (BSA) (1 mg/ml) for 4 h at 4°C. Beads were washed three times with PD150 (10 mM Tris pH 7.5, 150 mM NaCl, 0.1% Triton X-100, 20% glycerol, 1 mM DTT, protease inhibitor cocktail, PhosSTOP) to remove unbound peptides. Peptide-bound beads were incubated with nuclear extract for 2 h and washed four times with PD300. Bound proteins were eluted with SDS sample buffer, separated by SDS-PAGE and analysed by immunoblotting.

### Immunofluorescence

Cells were grown on coverslips, washed in PBS, fixed in 3.7% formaldehyde/PBS (15 min, 4°C) and permeabilised in 0.2% Triton X-100/PBS (5 min, 4°C). The coverslips were then washed in PBS, and blocked with 3% low-fat milk/PBS for 1 h at room temperature. Following overnight incubation with primary antibodies at 4°C, the coverslips were washed and incubated with secondary antibodies (Molecular Probes, Eugene, OR, USA) for 60 min at 37°C, and then washed again and counterstained with 4',6-diamidino-2-phenylindole (DAPI; 1 μg/μl, Vectashield, Vector Laboratories Inc., Burlingame, CA, USA)). Pictures were captured using epifluorescence microscopy (Leica DM6000B) using Leica Application Suite software.

### Fluorescence-activated cell sorting (FACS) analysis

C2C12 myoblasts were cultured in growing conditions and collected at 24 h, 48 h, 72 h and 96 h after plating. Cells were divided in aliquots of 1.2 × 10^6 ^cells per tube, washed with cold PBS 1 ×, fixed by 70% cold ethanol and incubated for 30 min on ice. After incubation, cells were washed with PBS 1 ×, resuspended in 0.5 ml of PBS 1 ×/RNase A (100 μg/ml) and incubated at 37°C for 30 min. Finally, propidium iodide (20 μg/ml) was added and the cells were incubated in the dark for 30 min at 4°C. The samples were then analyzed for the cell cycle profile and the cell death profile using a Becton Dickinson Instrument.

## Competing interests

The authors declare that they have no competing interests.

## Authors' contributions

LS, ZJ and CP designed and performed experiments, analyzed data and wrote the manuscript. AS performed *in vitro *experiments. BB performed experiments and analyzed data. DP performed chromatography experiments. RK and DS purified and provided histone peptides. CM performed experiments on satellite muscle cells. RM purified and provided Ezh1 Ab. PLP and KH participated in critically reviewing the data. WF conceived the study and analyzed *in vitro *data and participated in manuscript preparation. VO conceived the study and wrote the manuscript. All authors read and approved the final manuscript.

## Supplementary Material

Additional file 1 **Dynamics of PRC2 components in human skeletal muscle and satellite cells**. **(A) **Expression levels of *hEzh2*, *hEzh1 *and *hSuz12 *were measured by real-time PCR in human myoblasts grown in growth medium (GM) or differentiation medium (DM) (1 day, 4 days and 8 days after induction of differentiation). The transcription levels were normalised to *hGapdh *expression and represent the mean of three independent experiments ± SD. Fold enrichment was calculated in comparison to myoblasts in GM. **(B) **Expression levels of *Ezh2*, *Ezh1 *and *Suz12 *were measured by real-time PCR in myofibre-derived satellite cells grown in GM or DM (72 h after differentiation induction). *Pax7 *was used as a control for these cells and *myogenin *(*MyoG*) and *myosin heavy chain IIB *(*MHCIIB*) were used as muscle differentiation controls. The transcription levels were normalised to *Gapdh *expression and represent the mean of three independent experiments ± SD. Fold enrichment was calculated in comparison to myoblasts in GM. Click here for file

Additional file 2 **C2C12-H89 treatment impairs muscle gene activation**. **(A) **Schematic representation of the design of Msk1 inhibitor H89 treatment used in this study. **(B) **The effect of H89 treatment (5 μM and 10 μM) on C2C12 muscle cell differentiation was analysed in differentiation medium (DM) (48 h after treatment) by phase-contrast microscopy. **(C) **Expression levels of *myogenin *(*MyoG*) and *muscle creatine kinase *(*mCK*) were measured by real-time PCR in C2C12 myoblasts cultured in growth medium (GM) or DM (48 h after differentiation induction) with or without Msk1 inhibitor H89 (5 μM). Transcription levels were normalised to *Gapdh *expression. The data are shown as the average of three independent experiments, with error bars representing standard deviation. Fold enrichment was calculated in comparison to myoblasts in GM. **(D) **Immunoblot of MyoG and mCK from whole cell extracts of C2C12 myoblasts cultured in GM or DM (48 h after differentiation induction) with or without H89 (5 μM). β-Tubulin was used as a loading control. **(E) **Chromatin immunoprecipitation (ChIP) analyses of *MyoG *promoter, *mCK *enhancer and *mCK *promoter were performed on chromatin prepared from C2C12 cells cultured in GM or DM for 48 h after induction of differentiation, using histone H3 phosphorylation at serine 10 (H3S10ph) antibody. Levels of H3S10ph were normalised to histone H3 density. The precipitated DNA fragments were subjected to real-time PCR analysis. ChIP values are presented as relative enrichments to myoblasts. The values represent the mean ± SD of three independent experiments. Click here for file

Additional file 3 **Efficiency of Ezh1 and Ezh2 small interfering RNA (siRNA) in C2C12 cells**. **(A) **Myoblasts were transfected with non-targeting siRNA (Nc = negative control) or siRNA against Ezh1 (siEzh1 no. 1 and siEzh1 no. 2), and the efficiency of siRNA was tested by real-time PCR in growth medium (GM) and in differentiation medium (DM) (48 h after differentiation induction). The transcription levels were normalised to *Gapdh *expression and represented as the average of three independent experiments ± SD. Fold enrichment was calculated in comparison to the negative control siRNA in GM. **(B) **Immunofluorescence for Ezh1 performed after delivery of siRNA into cells. Note the weak labelling in a high number of cells treated with Ezh1 siRNA (no. 2). Scale bar = 50 μm. **(C) **Myoblasts were transfected with non-targeting siRNA (Nc = negative control) or siRNA against Ezh2 (siEzh2 no. 1 and siEzh2 no. 2) and the efficiency of siRNA was tested by real-time PCR in GM and in DM (48 h after differentiation induction). The transcription levels were normalised to *Gapdh *expression and represented as the average of three independent experiments ± SD. Fold enrichment was calculated in comparison to the negative control siRNA in GM. **(D) **Immunofluorescence of Ezh2 48 h post transfection with siRNA (oligo no. 2). Scale bar = 100 μm. Click here for file

Additional file 4 **Ezh1-depleted human myoblasts and satellite cells show a delay in *myogenin *(*MyoG*) transcriptional activation**. **(A) **Human myoblasts were transfected with either non-targeting small interfering RNA (siRNA) (Nc = negative control) or siRNA against *Ezh1*. The effect of siRNA on cell morphology was analysed in growth medium (GM) (48 h after transfection) and in differentiation medium (DM) (2 and 4 days after differentiation induction) by phase-contrast microscopy. **(B) **The efficiency of siRNA for *Ezh1 *and the expression levels of *MyoG *were tested by real-time PCR in GM and in DM (2 days and 4 days after differentiation induction), in human myoblasts depleted for *Ezh1*. The transcription levels were normalised to *Gapdh *expression and are represented as the average of three independent experiments ± SD. Fold enrichment was calculated in comparison to negative control siRNA in GM. **(C) **Myofibre-derived satellite cells were transfected with non-targeting siRNA (Nc = negative control) or siRNA against *Ezh1 *(no. 1). The effect of siRNA on cell morphology was analysed in GM (48 h after transfection) and in DM (2 days after differentiation induction) by phase-contrast microscopy. **(D) **The efficiency of siRNA for *Ezh1 *and the expression levels of *MyoG *were tested by real-time PCR in GM and in DM (48 h after differentiation induction) in myofibre-derived satellite cells depleted for *Ezh1 *(oligo no. 1). The transcription levels were normalised to *Gapdh *expression. Fold enrichment was calculated as a percentage (%) of the negative control siRNA in GM. Click here for file

Additional file 5 **Suz12 small interfering RNA (siRNA) affects *myogenin *(*MyoG*) transcriptional activation in human myoblasts and in satellite cells**. **(A) **Human myoblasts were transfected either with non-targeting siRNA (Nc = negative control) or siRNA against *Suz12*. The effect of siRNA on cell morphology was analysed in growth medium (GM) (48 h after transfection) and in differentiation medium (DM) (2 and 4 days after differentiation induction) by phase-contrast microscopy. **(B) **The efficiency of siRNA for *Suz12 *and the expression levels of *MyoG *were tested by real-time PCR in GM and in DM (2 days and 4 days after differentiation induction), in human myoblasts depleted for *Suz12*. The transcription levels were normalised to *Gapdh *expression and are represented as the average of three independent experiments ± SD. Fold enrichment was calculated in comparison to the negative control siRNA in GM. **(C) **Myofibre-derived satellite cells were transfected with either non-targeting siRNA (Nc = negative control) or siRNA against Suz12 (no. 1). The effect of siRNA on cell morphology was analysed in GM (48 h after transfection) and in DM (2 days after differentiation induction) by phase-contrast microscopy. **(D) **The efficiency of siRNA for Suz12 and the expression levels of *MyoG *were tested by real-time PCR in GM and in DM (2 days after differentiation induction) in myofibre-derived satellite cells depleted for Suz12. The transcription levels were normalised to *Gapdh *expression. Fold enrichment was calculated in comparison to the negative control siRNA in GM. Click here for file
