# Supplementary figures and images for: Chromatin regulated interchange between polycomb repressive complex 2 (PRC2)-Ezh2 and PRC2-Ezh1 complexes controls myogenin activation in skeletal muscle cells
Source: Epigenetics Chromatin. 2011 Sep 5;4:16. doi: 10.1186/1756-8935-4-16 (PMC3180244; doi:10.1186/1756-8935-4-16)

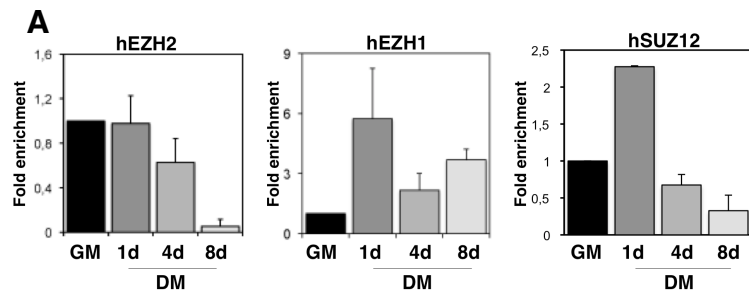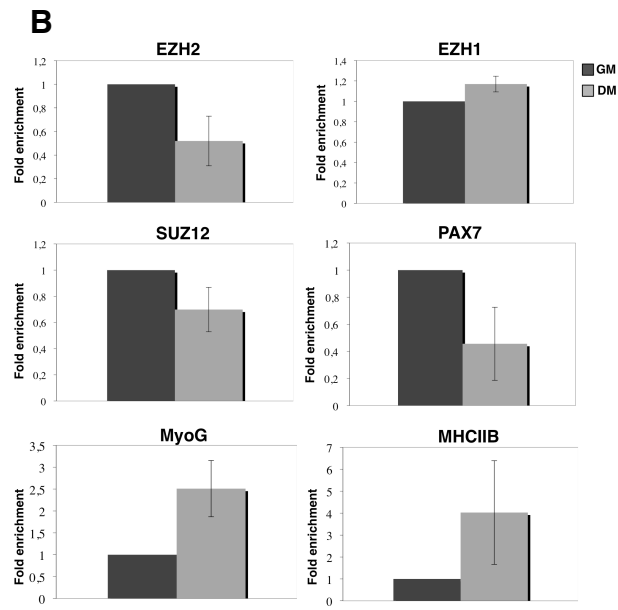

Supplement: Additional file 1 — Dynamics of PRC2 components in human skeletal muscle and satellite cells. (A) Expression levels of hEzh2, hEzh1 and hSuz12 were measured by real-time PCR in human myoblasts grown in growth medium (GM) or differentiation medium (DM) (1 day, 4 days and 8 days after induction of differentiation). The transcription levels were normalised to hGapdh expression and represent the mean of three independent experiments ± SD. Fold enrichment was calculated in comparison to myoblasts in GM. (B) Expression levels of Ezh2, Ezh1 and Suz12 were measured by real-time PCR in myofibre-derived satellite cells grown in GM or DM (72 h after differentiation induction). Pax7 was used as a control for these cells and myogenin (MyoG) and myosin heavy chain IIB (MHCIIB) were used as muscle differentiation controls. The transcription levels were normalised to Gapdh expression and represent the mean of three independent experiments ± SD. Fold enrichment was calculated in comparison to myoblasts in GM. [file 1756-8935-4-16-S1.PDF]

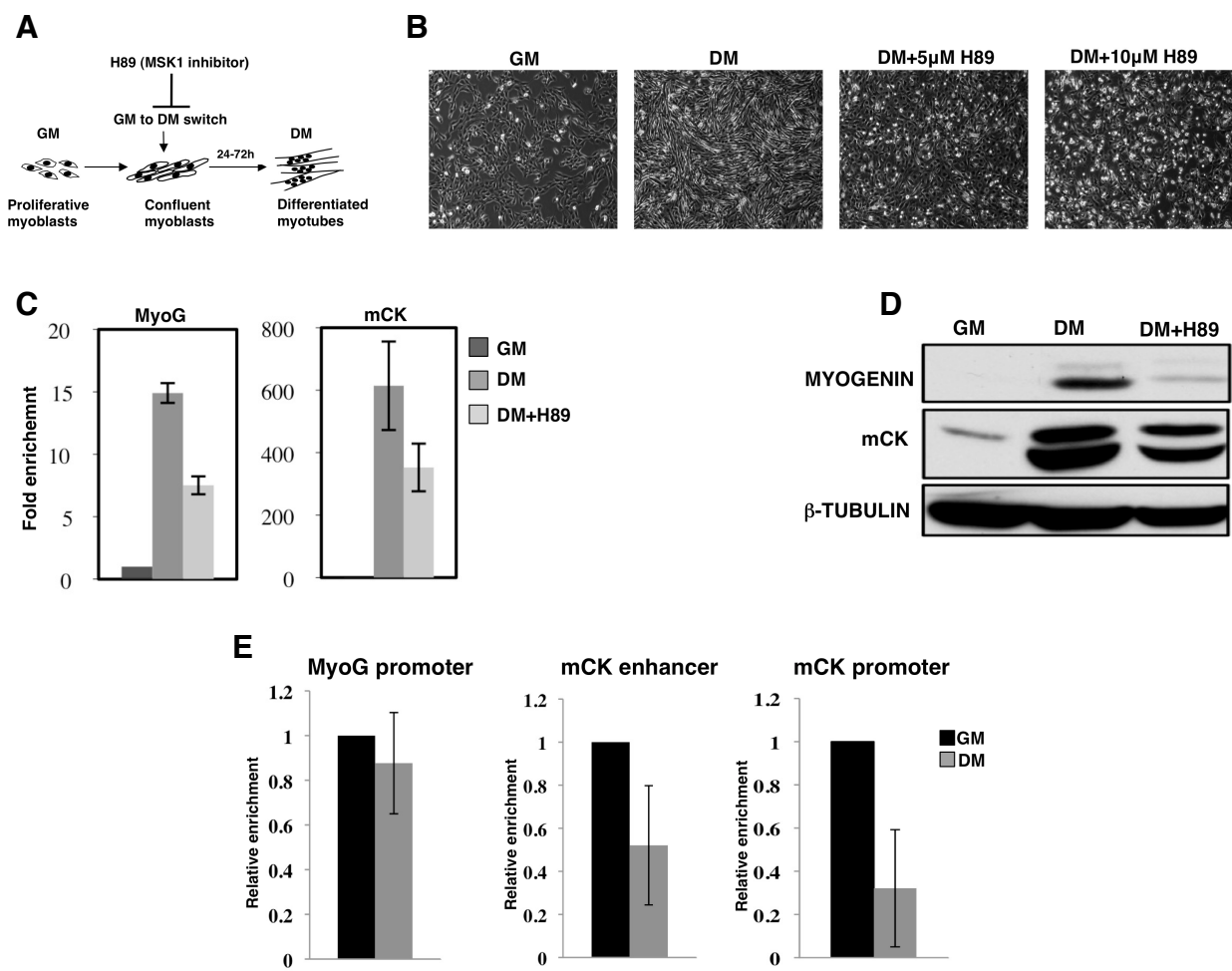

Supplement: Additional file 2 — C2C12-H89 treatment impairs muscle gene activation. (A) Schematic representation of the design of Msk1 inhibitor H89 treatment used in this study. (B) The effect of H89 treatment (5 μM and 10 μM) on C2C12 muscle cell differentiation was analysed in differentiation medium (DM) (48 h after treatment) by phase-contrast microscopy. (C) Expression levels of myogenin (MyoG) and muscle creatine kinase (mCK) were measured by real-time PCR in C2C12 myoblasts cultured in growth medium (GM) or DM (48 h after differentiation induction) with or without Msk1 inhibitor H89 (5 μM). Transcription levels were normalised to Gapdh expression. The data are shown as the average of three independent experiments, with error bars representing standard deviation. Fold enrichment was calculated in comparison to myoblasts in GM. (D) Immunoblot of MyoG and mCK from whole cell extracts of C2C12 myoblasts cultured in GM or DM (48 h after differentiation induction) with or without H89 (5 μM). β-Tubulin was used as a loading control. (E) Chromatin immunoprecipitation (ChIP) analyses of MyoG promoter, mCK enhancer and mCK promoter were performed on chromatin prepared from C2C12 cells cultured in GM or DM for 48 h after induction of differentiation, using histone H3 phosphorylation at serine 10 (H3S10ph) antibody. Levels of H3S10ph were normalised to histone H3 density. The precipitated DNA fragments were subjected to real-time PCR analysis. ChIP values are presented as relative enrichments to myoblasts. The values represent the mean ± SD of three independent experiments. [file 1756-8935-4-16-S2.PDF]

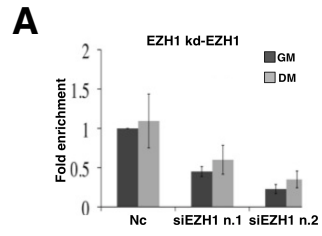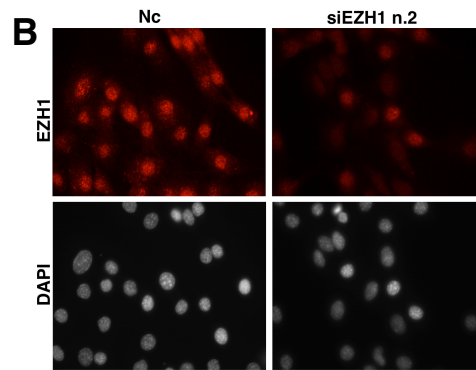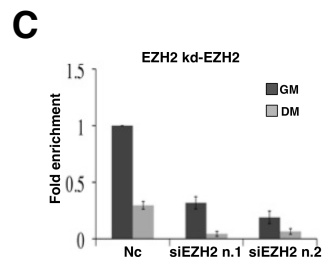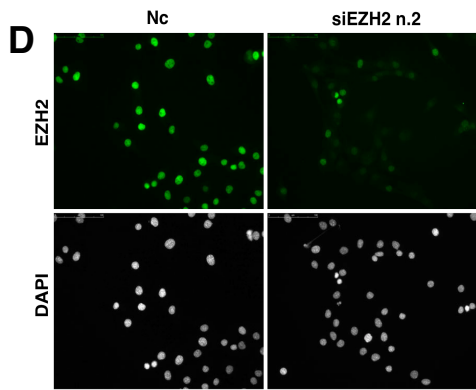

Supplement: Additional file 3 — Efficiency of Ezh1 and Ezh2 small interfering RNA (siRNA) in C2C12 cells. (A) Myoblasts were transfected with non-targeting siRNA (Nc = negative control) or siRNA against Ezh1 (siEzh1 no. 1 and siEzh1 no. 2), and the efficiency of siRNA was tested by real-time PCR in growth medium (GM) and in differentiation medium (DM) (48 h after differentiation induction). The transcription levels were normalised to Gapdh expression and represented as the average of three independent experiments ± SD. Fold enrichment was calculated in comparison to the negative control siRNA in GM. (B) Immunofluorescence for Ezh1 performed after delivery of siRNA into cells. Note the weak labelling in a high number of cells treated with Ezh1 siRNA (no. 2). Scale bar = 50 μm. (C) Myoblasts were transfected with non-targeting siRNA (Nc = negative control) or siRNA against Ezh2 (siEzh2 no. 1 and siEzh2 no. 2) and the efficiency of siRNA was tested by real-time PCR in GM and in DM (48 h after differentiation induction). The transcription levels were normalised to Gapdh expression and represented as the average of three independent experiments ± SD. Fold enrichment was calculated in comparison to the negative control siRNA in GM. (D) Immunofluorescence of Ezh2 48 h post transfection with siRNA (oligo no. 2). Scale bar = 100 μm. [file 1756-8935-4-16-S3.PDF]

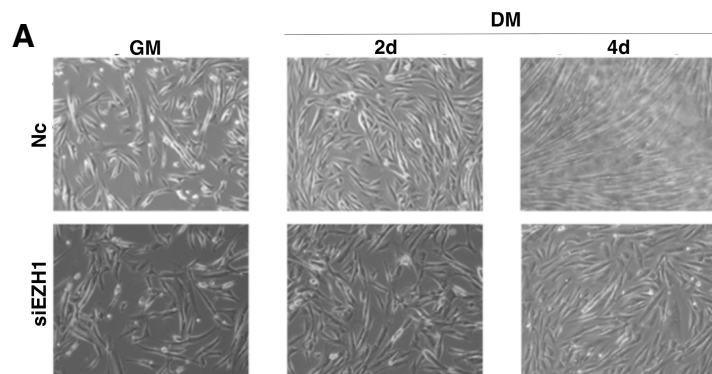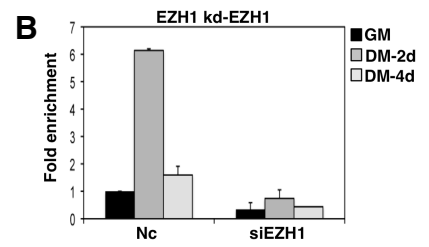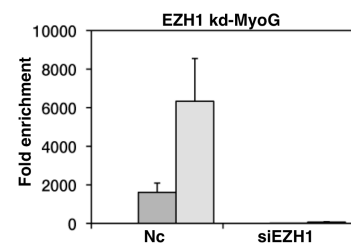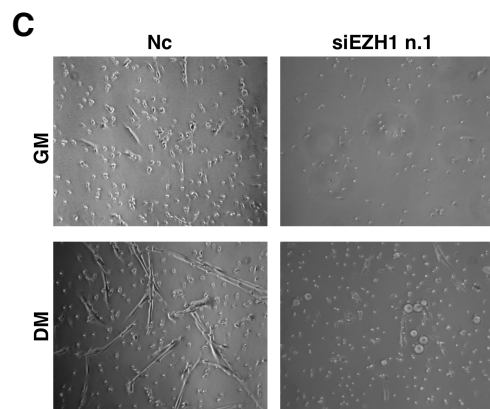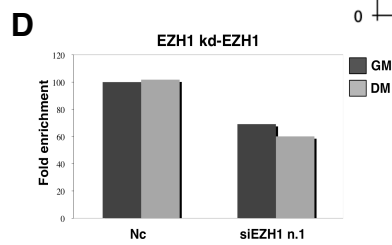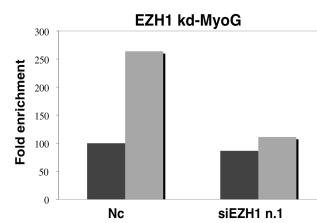

Supplement: Additional file 4 — Ezh1-depleted human myoblasts and satellite cells show a delay in myogenin (MyoG) transcriptional activation. (A) Human myoblasts were transfected with either non-targeting small interfering RNA (siRNA) (Nc = negative control) or siRNA against Ezh1. The effect of siRNA on cell morphology was analysed in growth medium (GM) (48 h after transfection) and in differentiation medium (DM) (2 and 4 days after differentiation induction) by phase-contrast microscopy. (B) The efficiency of siRNA for Ezh1 and the expression levels of MyoG were tested by real-time PCR in GM and in DM (2 days and 4 days after differentiation induction), in human myoblasts depleted for Ezh1. The transcription levels were normalised to Gapdh expression and are represented as the average of three independent experiments ± SD. Fold enrichment was calculated in comparison to negative control siRNA in GM. (C) Myofibre-derived satellite cells were transfected with non-targeting siRNA (Nc = negative control) or siRNA against Ezh1 (no. 1). The effect of siRNA on cell morphology was analysed in GM (48 h after transfection) and in DM (2 days after differentiation induction) by phase-contrast microscopy. (D) The efficiency of siRNA for Ezh1 and the expression levels of MyoG were tested by real-time PCR in GM and in DM (48 h after differentiation induction) in myofibre-derived satellite cells depleted for Ezh1 (oligo no. 1). The transcription levels were normalised to Gapdh expression. Fold enrichment was calculated as a percentage (%) of the negative control siRNA in GM. [file 1756-8935-4-16-S4.PDF]

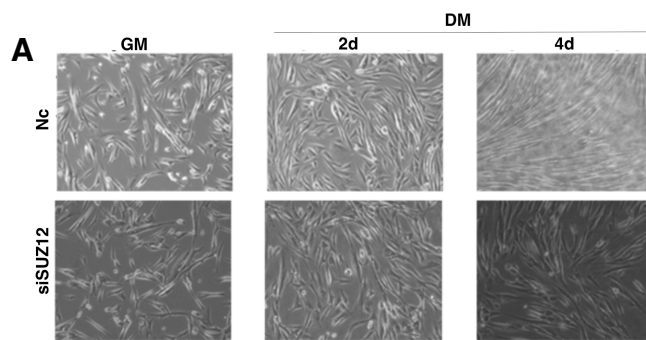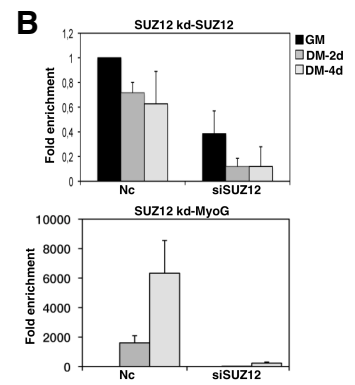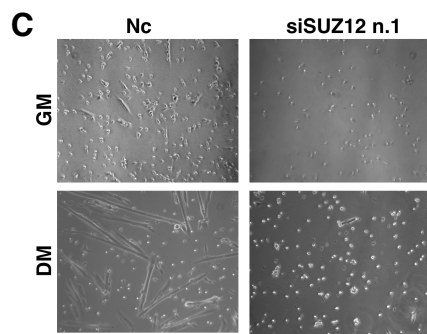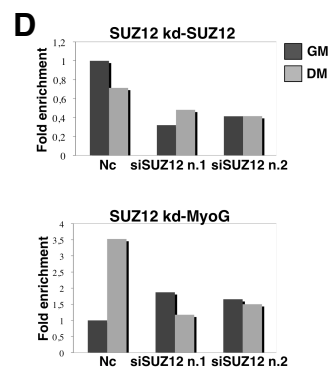

Supplement: Additional file 5 — Suz12 small interfering RNA (siRNA) affects myogenin (MyoG) transcriptional activation in human myoblasts and in satellite cells. (A) Human myoblasts were transfected either with non-targeting siRNA (Nc = negative control) or siRNA against Suz12. The effect of siRNA on cell morphology was analysed in growth medium (GM) (48 h after transfection) and in differentiation medium (DM) (2 and 4 days after differentiation induction) by phase-contrast microscopy. (B) The efficiency of siRNA for Suz12 and the expression levels of MyoG were tested by real-time PCR in GM and in DM (2 days and 4 days after differentiation induction), in human myoblasts depleted for Suz12. The transcription levels were normalised to Gapdh expression and are represented as the average of three independent experiments ± SD. Fold enrichment was calculated in comparison to the negative control siRNA in GM. (C) Myofibre-derived satellite cells were transfected with either non-targeting siRNA (Nc = negative control) or siRNA against Suz12 (no. 1). The effect of siRNA on cell morphology was analysed in GM (48 h after transfection) and in DM (2 days after differentiation induction) by phase-contrast microscopy. (D) The efficiency of siRNA for Suz12 and the expression levels of MyoG were tested by real-time PCR in GM and in DM (2 days after differentiation induction) in myofibre-derived satellite cells depleted for Suz12. The transcription levels were normalised to Gapdh expression. Fold enrichment was calculated in comparison to the negative control siRNA in GM. [file 1756-8935-4-16-S5.PDF]
